# 3-(2-Hydroxy­phen­yl)-5-(2-methoxy­phenyl)-1*H*-pyrazole

**DOI:** 10.1107/S1600536808025725

**Published:** 2008-08-16

**Authors:** Aurangzeb Hasan, Sumera Ikram, Amir Badshah, Michael Bolte, Mehwash Zia

**Affiliations:** a Department of Chemistry, Quaid-i-Azam University, Islamabad 45320, Pakistan; b Institut für Anorganische Chemie, J. W. Goethe-Universität, Max-von-Laue-Strasse 7, 60438 Frankfurt/Main, Germany

## Abstract

The title compound, C_16_H_14_N_2_O_2_, was derived from 1-(2-hydroxy­phen­yl)-3-(2-methoxy­phen­yl)propane-1,3-dione. The mol­ecule is essentially planar (r.m.s. deviation for all non-H atoms = 0.089 Å). Two intra­molecular hydrogen bonds stabilize the mol­ecular conformation and one N—H⋯O hydrogen bond stabilizes the crystal structure.

## Related literature

For related literature, see: Ahmad *et al.* (1990 ▶, 1997 ▶); Ezava *et al.* (2005 ▶); Feierman & Cederbaum (1986 ▶); Sanz *et al.* (1998 ▶); Alcaraz *et al.* (1993 ▶); Hamper *et al.* (1997 ▶); Fujio (1999 ▶).
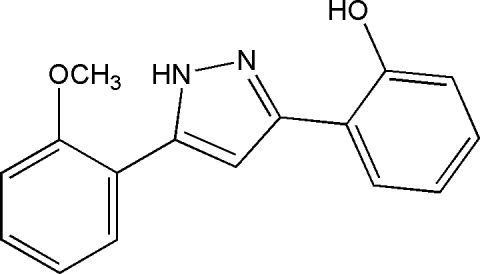


## Experimental

### 

#### Crystal data

C_16_H_14_N_2_O_2_
*M* *_r_* = 266.29
Orthorhombic, 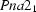

*a* = 17.5626 (15) Å
*b* = 10.2239 (7) Å
*c* = 7.4513 (7) Å
*V* = 1337.94 (19) Å^3^
*Z* = 4
Mo *K*α radiation
μ = 0.09 mm^−1^
*T* = 173 (2) K
0.27 × 0.25 × 0.24 mm

#### Data collection

Stoe IPDSII two-circle diffractometer
Absorption correction: none
10969 measured reflections
1777 independent reflections
1620 reflections with *I* > 2σ(*I*)
*R*_int_ = 0.057

#### Refinement

*R*[*F* ^2^ > 2σ(*F* ^2^)] = 0.034
*wR*(*F* ^2^) = 0.090
*S* = 1.03
1777 reflections
191 parameters
1 restraint
H atoms treated by a mixture of independent and constrained refinement
Δρ_max_ = 0.18 e Å^−3^
Δρ_min_ = −0.16 e Å^−3^

### 

Data collection: *X-AREA* (Stoe & Cie, 2001 ▶); cell refinement: *X-AREA*; data reduction: *X-AREA*; program(s) used to solve structure: *SHELXS97* (Sheldrick, 2008 ▶); program(s) used to refine structure: *SHELXL97* (Sheldrick, 2008 ▶); molecular graphics: *XP* in *SHELXTL-Plus* (Sheldrick, 2008 ▶); software used to prepare material for publication: *SHELXL97*.

## Supplementary Material

Crystal structure: contains datablocks I, global. DOI: 10.1107/S1600536808025725/bx2163sup1.cif

Structure factors: contains datablocks I. DOI: 10.1107/S1600536808025725/bx2163Isup2.hkl

Additional supplementary materials:  crystallographic information; 3D view; checkCIF report

## Figures and Tables

**Table 1:** Hydrogen-bond geometry (Å, °)

*D*—H⋯*A*	*D*—H	H⋯*A*	*D*⋯*A*	*D*—H⋯*A*
O2—H2⋯N2	0.99 (4)	1.64 (4)	2.560 (2)	152 (3)
N1—H1⋯O1	0.92 (3)	2.07 (3)	2.628 (2)	118 (2)
N1—H1⋯O2^i^	0.92 (3)	2.09 (3)	2.892 (2)	146 (3)

Symmetry code: (i) 
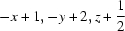
.

**Fig. 1.**
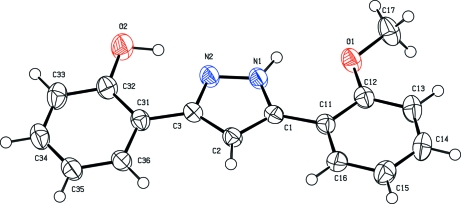
Perspective view of the title compound with the atom numbering scheme; displacement ellipsoids are at the 50% probability level; H atoms are drawn as small spheres of arbitrary radii.

## supplementary crystallographic information

### Comment

3,5-substituted Pyrazoles are important class of compounds.These have been proven to be a selective inhibitor of COX in isoenzyme in human blood and are used for the development of anti-inflamatory drugs and analgesic medicines (Ezava *et al.*, 2005). Disubstituted pyrazoles have been reported as an important intermediate in the synthesis of herbicides (US patent 5191087, 1993; US patent 5698708, 1997) and for the treatmet of pain and disorders such as Arthritis (US patent 5908857, 1999).Pyrazoles are inhibitors of alchol dehydrogenase and have been found to be effective inhibitors for the oxidation of ethanol by liver microsomes (Feierman & Cederbaum, 1986). 3,5-disubstituted pyrazoles are also uesd to form solid dinuclear complexes (Sanz *et al.*, 1998). The molecule is essentially planar (r.m.s. deviation for all non-H atoms 0.089 Å). Two intramolecular hydrogen bonds stabilize the molecular conformation and one N—H···O hydrogen bond is stabilizing the crystal structure.

### Experimental

1-(2'-hydroxyphenyl)-3-(2''-methoxyphenyl) propane-1,3-dione was prepared by a modified Baker Venkataram rearrangement as reported earlier (Ahmad *et al.*, 1997). Purification was carried out by recrystallization using absolute ethanol. 1-*H*-3(2-hydroxyphenyl)-5-(2-methoxyphenyl) pyrazole was synthesized by reacting hydrazine hydrate (0.5 g, 10 mmol) with 1-(2-hydroxyphenyl)-3-(2-methoxyphenyl) propane-1,3-dione (2.7 g, 10 mmol) in 100 ml of absolute ethanol. The mixture was refluxed for seven hours. Solvent was removed under reduced pressure. Compound (II) was synthesized by adding 0.1 mole of phenyl hydrazine in 0.1 mole of compound (II) dissolved in 200 ml of absolute ethanol. The mixture was refluxed for 7 h. Solvent was removed under reduced pressure. Highly viscous residue was recrystallized using absolute ethanol. (Yield: 96%, m.p: 456k)

### Refinement

In the absence of anomalous scatterers 1544 Friedel pairs were merged. H atoms were located in a difference map, but those bonded to C were geometrically positioned and refined using a riding model with fixed individual displacement parameters [U(H) = 1.2 U_eq_(C) or U(H) = 1.5 U_eq_(C_methyl_)] and with C—H = 0.95 Å or C_methyl_—H  = 0.98 Å. The methyl group was allowed to rotate but not to tip. The H atoms bonded to N and O were freely refined.

### Crystal data

**Table:**

C_16_H_14_N_2_O_2_	*D*_x_ = 1.322 Mg m^−^^3^
*M**_r_* = 266.29	Melting point: 456 K
Orthorhombic, *P**n**a*2_1_	Mo *K*α radiation λ = 0.71073 Å
Hall symbol: P 2c -2n	Cell parameters from 11915 reflections
*a* = 17.5626 (15) Å	θ = 3.4–29.6º
*b* = 10.2239 (7) Å	µ = 0.09 mm^−^^1^
*c* = 7.4513 (7) Å	*T* = 173 (2) K
*V* = 1337.94 (19) Å^3^	Block, light yellow
*Z* = 4	0.27 × 0.25 × 0.24 mm
*F*_000_ = 560	

### Data collection

**Table:**

Stoe IPDSII two-circle diffractometer	1620 reflections with *I* > 2σ(*I*)
Radiation source: fine-focus sealed tube	*R*_int_ = 0.057
Monochromator: graphite	θ_max_ = 28.3º
*T* = 173(2) K	θ_min_ = 3.6º
ω scans	*h* = −20→23
Absorption correction: none	*k* = −11→13
10969 measured reflections	*l* = −8→9
1777 independent reflections	

### Refinement

**Table:**

Refinement on *F*^2^	Secondary atom site location: difference Fourier map
Least-squares matrix: full	Hydrogen site location: inferred from neighbouring sites
*R*[*F*^2^ > 2σ(*F*^2^)] = 0.034	H atoms treated by a mixture of independent and constrained refinement
*wR*(*F*^2^) = 0.091	*w* = 1/[σ^2^(*F*_o_^2^) + (0.0644*P*)^2^ + 0.0395*P*] where *P* = (*F*_o_^2^ + 2*F*_c_^2^)/3
*S* = 1.03	(Δ/σ)_max_ < 0.001
1777 reflections	Δρ_max_ = 0.18 e Å^−^^3^
191 parameters	Δρ_min_ = −0.16 e Å^−^^3^
1 restraint	Extinction correction: SHELXL97 (Sheldrick, 2008), Fc^*^=kFc[1+0.001xFc^2^λ^3^/sin(2θ)]^-1/4^
Primary atom site location: structure-invariant direct methods	Extinction coefficient: 0.049 (6)

### Special details

**Table:**

Geometry. All e.s.d.'s (except the e.s.d. in the dihedral angle between two l.s. planes) are estimated using the full covariance matrix. The cell e.s.d.'s are taken into account individually in the estimation of e.s.d.'s in distances, angles and torsion angles; correlations between e.s.d.'s in cell parameters are only used when they are defined by crystal symmetry. An approximate (isotropic) treatment of cell e.s.d.'s is used for estimating e.s.d.'s involving l.s. planes.
Refinement. Refinement of *F*^2^ against ALL reflections. The weighted *R*-factor *wR* and goodness of fit *S* are based on *F*^2^, conventional *R*-factors *R* are based on *F*, with *F* set to zero for negative *F*^2^. The threshold expression of *F*^2^ > σ(*F*^2^) is used only for calculating *R*-factors(gt) *etc*. and is not relevant to the choice of reflections for refinement. *R*-factors based on *F*^2^ are statistically about twice as large as those based on *F*, and *R*- factors based on ALL data will be even larger.

### Fractional atomic coordinates and isotropic or equivalent isotropic displacement parameters (Å^2^)

**Table:**

	*x*	*y*	*z*	*U*_iso_*/*U*_eq_	
O1	0.49830 (7)	0.72482 (13)	0.76358 (19)	0.0401 (3)	
O2	0.42612 (9)	1.04511 (15)	0.0584 (2)	0.0507 (4)	
H2	0.441 (2)	0.992 (4)	0.165 (6)	0.093 (11)*	
N1	0.44362 (8)	0.80017 (15)	0.4520 (2)	0.0340 (3)	
H1	0.4833 (15)	0.825 (3)	0.525 (4)	0.058 (7)*	
N2	0.43083 (8)	0.86611 (15)	0.2984 (2)	0.0369 (3)	
C1	0.39489 (9)	0.69783 (15)	0.4736 (2)	0.0289 (3)	
C2	0.34823 (9)	0.69776 (15)	0.3232 (2)	0.0306 (3)	
H2A	0.3083	0.6381	0.2968	0.037*	
C3	0.37225 (9)	0.80434 (16)	0.2179 (2)	0.0304 (3)	
C11	0.39509 (9)	0.61110 (16)	0.6314 (2)	0.0306 (3)	
C12	0.44651 (9)	0.62379 (17)	0.7757 (2)	0.0339 (3)	
C13	0.44373 (11)	0.5373 (2)	0.9203 (3)	0.0425 (4)	
H13	0.4788	0.5461	1.0165	0.051*	
C14	0.38945 (12)	0.4381 (2)	0.9235 (3)	0.0461 (5)	
H14	0.3882	0.3786	1.0214	0.055*	
C15	0.33733 (11)	0.42539 (19)	0.7852 (3)	0.0437 (4)	
H15	0.2998	0.3585	0.7891	0.052*	
C16	0.34031 (10)	0.51098 (17)	0.6410 (3)	0.0353 (4)	
H16	0.3045	0.5018	0.5463	0.042*	
C17	0.55036 (12)	0.7438 (3)	0.9090 (3)	0.0520 (5)	
H17A	0.5825	0.6660	0.9222	0.078*	
H17B	0.5218	0.7583	1.0202	0.078*	
H17C	0.5825	0.8201	0.8843	0.078*	
C31	0.34524 (9)	0.85273 (17)	0.0429 (2)	0.0314 (3)	
C32	0.37398 (10)	0.96997 (18)	−0.0313 (3)	0.0367 (4)	
C33	0.34958 (11)	1.0130 (2)	−0.1993 (3)	0.0438 (4)	
H33	0.3693	1.0919	−0.2482	0.053*	
C34	0.29652 (12)	0.9407 (2)	−0.2952 (3)	0.0437 (4)	
H34	0.2805	0.9700	−0.4102	0.052*	
C35	0.26648 (11)	0.82574 (19)	−0.2245 (3)	0.0417 (4)	
H35	0.2297	0.7772	−0.2900	0.050*	
C36	0.29090 (10)	0.78266 (17)	−0.0569 (2)	0.0354 (4)	
H36	0.2704	0.7041	−0.0089	0.042*	

### Atomic displacement parameters (Å^2^)

**Table:**

	*U*^11^	*U*^22^	*U*^33^	*U*^12^	*U*^13^	*U*^23^
O1	0.0371 (6)	0.0507 (8)	0.0327 (6)	−0.0037 (5)	−0.0084 (5)	0.0025 (6)
O2	0.0531 (8)	0.0541 (8)	0.0448 (8)	−0.0219 (7)	−0.0050 (7)	0.0142 (7)
N1	0.0363 (7)	0.0385 (7)	0.0273 (7)	−0.0070 (6)	−0.0052 (6)	0.0026 (6)
N2	0.0390 (7)	0.0402 (7)	0.0314 (8)	−0.0087 (6)	−0.0060 (6)	0.0038 (6)
C1	0.0298 (7)	0.0302 (7)	0.0268 (8)	0.0005 (6)	−0.0005 (6)	−0.0037 (6)
C2	0.0306 (7)	0.0329 (7)	0.0283 (8)	−0.0029 (6)	−0.0024 (6)	−0.0014 (6)
C3	0.0304 (7)	0.0336 (8)	0.0271 (8)	−0.0008 (6)	−0.0013 (6)	−0.0018 (6)
C11	0.0325 (7)	0.0330 (7)	0.0263 (7)	0.0057 (6)	0.0020 (6)	−0.0017 (6)
C12	0.0325 (7)	0.0394 (8)	0.0299 (8)	0.0063 (6)	0.0008 (6)	−0.0018 (7)
C13	0.0413 (9)	0.0537 (11)	0.0326 (9)	0.0113 (8)	−0.0018 (7)	0.0071 (8)
C14	0.0493 (10)	0.0513 (11)	0.0378 (10)	0.0074 (8)	0.0033 (8)	0.0156 (9)
C15	0.0476 (9)	0.0410 (9)	0.0426 (11)	−0.0005 (7)	0.0046 (8)	0.0065 (8)
C16	0.0389 (8)	0.0333 (8)	0.0337 (9)	0.0004 (7)	0.0005 (7)	−0.0010 (7)
C17	0.0431 (10)	0.0746 (14)	0.0384 (11)	−0.0055 (10)	−0.0137 (8)	0.0031 (10)
C31	0.0314 (7)	0.0365 (8)	0.0263 (8)	0.0041 (6)	0.0016 (6)	−0.0006 (6)
C32	0.0357 (8)	0.0434 (9)	0.0310 (9)	−0.0011 (7)	0.0048 (7)	0.0015 (7)
C33	0.0483 (10)	0.0488 (10)	0.0342 (10)	0.0069 (8)	0.0084 (8)	0.0103 (8)
C34	0.0510 (10)	0.0530 (10)	0.0271 (8)	0.0189 (9)	−0.0001 (7)	0.0001 (8)
C35	0.0464 (9)	0.0464 (9)	0.0324 (9)	0.0122 (7)	−0.0083 (7)	−0.0067 (8)
C36	0.0384 (8)	0.0370 (8)	0.0307 (8)	0.0036 (7)	−0.0040 (7)	−0.0037 (7)

### Geometric parameters (Å, °)

**Table:**

O1—C12	1.379 (2)	C14—C15	1.384 (3)
O1—C17	1.431 (2)	C14—H14	0.9500
O2—C32	1.369 (2)	C15—C16	1.387 (3)
O2—H2	0.99 (4)	C15—H15	0.9500
N1—N2	1.347 (2)	C16—H16	0.9500
N1—C1	1.361 (2)	C17—H17A	0.9800
N1—H1	0.92 (3)	C17—H17B	0.9800
N2—C3	1.348 (2)	C17—H17C	0.9800
C1—C2	1.388 (2)	C31—C36	1.406 (2)
C1—C11	1.473 (2)	C31—C32	1.413 (2)
C2—C3	1.408 (2)	C32—C33	1.395 (3)
C2—H2A	0.9500	C33—C34	1.387 (3)
C3—C31	1.473 (2)	C33—H33	0.9500
C11—C16	1.407 (2)	C34—C35	1.392 (3)
C11—C12	1.410 (2)	C34—H34	0.9500
C12—C13	1.394 (3)	C35—C36	1.392 (3)
C13—C14	1.392 (3)	C35—H35	0.9500
C13—H13	0.9500	C36—H36	0.9500
			
C12—O1—C17	118.24 (16)	C16—C15—H15	120.2
C32—O2—H2	105 (2)	C15—C16—C11	121.61 (18)
N2—N1—C1	112.35 (14)	C15—C16—H16	119.2
N2—N1—H1	119.2 (18)	C11—C16—H16	119.2
C1—N1—H1	128.3 (18)	O1—C17—H17A	109.5
N1—N2—C3	105.72 (14)	O1—C17—H17B	109.5
N1—C1—C2	106.03 (15)	H17A—C17—H17B	109.5
N1—C1—C11	123.76 (15)	O1—C17—H17C	109.5
C2—C1—C11	130.20 (14)	H17A—C17—H17C	109.5
C1—C2—C3	105.82 (14)	H17B—C17—H17C	109.5
C1—C2—H2A	127.1	C36—C31—C32	117.91 (16)
C3—C2—H2A	127.1	C36—C31—C3	121.03 (15)
N2—C3—C2	110.08 (15)	C32—C31—C3	121.05 (16)
N2—C3—C31	118.85 (15)	O2—C32—C33	117.80 (17)
C2—C3—C31	131.07 (15)	O2—C32—C31	121.61 (17)
C16—C11—C12	117.79 (16)	C33—C32—C31	120.59 (18)
C16—C11—C1	118.49 (15)	C34—C33—C32	120.02 (18)
C12—C11—C1	123.71 (15)	C34—C33—H33	120.0
O1—C12—C13	123.28 (17)	C32—C33—H33	120.0
O1—C12—C11	116.16 (15)	C33—C34—C35	120.64 (18)
C13—C12—C11	120.56 (17)	C33—C34—H34	119.7
C14—C13—C12	119.95 (18)	C35—C34—H34	119.7
C14—C13—H13	120.0	C36—C35—C34	119.34 (18)
C12—C13—H13	120.0	C36—C35—H35	120.3
C15—C14—C13	120.55 (18)	C34—C35—H35	120.3
C15—C14—H14	119.7	C35—C36—C31	121.49 (17)
C13—C14—H14	119.7	C35—C36—H36	119.3
C14—C15—C16	119.51 (18)	C31—C36—H36	119.3
C14—C15—H15	120.2		
			
C1—N1—N2—C3	−0.2 (2)	C12—C13—C14—C15	−0.9 (3)
N2—N1—C1—C2	0.46 (19)	C13—C14—C15—C16	1.2 (3)
N2—N1—C1—C11	−178.61 (14)	C14—C15—C16—C11	−0.1 (3)
N1—C1—C2—C3	−0.50 (18)	C12—C11—C16—C15	−1.2 (3)
C11—C1—C2—C3	178.48 (16)	C1—C11—C16—C15	179.75 (16)
N1—N2—C3—C2	−0.1 (2)	N2—C3—C31—C36	172.07 (16)
N1—N2—C3—C31	−179.01 (14)	C2—C3—C31—C36	−6.5 (3)
C1—C2—C3—N2	0.4 (2)	N2—C3—C31—C32	−7.0 (2)
C1—C2—C3—C31	179.10 (16)	C2—C3—C31—C32	174.43 (18)
N1—C1—C11—C16	178.05 (15)	C36—C31—C32—O2	178.95 (17)
C2—C1—C11—C16	−0.8 (3)	C3—C31—C32—O2	−2.0 (3)
N1—C1—C11—C12	−0.9 (2)	C36—C31—C32—C33	−0.8 (2)
C2—C1—C11—C12	−179.75 (17)	C3—C31—C32—C33	178.21 (15)
C17—O1—C12—C13	−1.8 (3)	O2—C32—C33—C34	−179.68 (18)
C17—O1—C12—C11	178.37 (16)	C31—C32—C33—C34	0.1 (3)
C16—C11—C12—O1	−178.60 (15)	C32—C33—C34—C35	0.7 (3)
C1—C11—C12—O1	0.4 (2)	C33—C34—C35—C36	−0.8 (3)
C16—C11—C12—C13	1.5 (2)	C34—C35—C36—C31	0.1 (3)
C1—C11—C12—C13	−179.50 (16)	C32—C31—C36—C35	0.8 (2)
O1—C12—C13—C14	179.62 (17)	C3—C31—C36—C35	−178.30 (16)
C11—C12—C13—C14	−0.5 (3)		

### Hydrogen-bond geometry (Å, °)

**Table:**

*D*—H···*A*	*D*—H	H···*A*	*D*···*A*	*D*—H···*A*
O2—H2···N2	0.99 (4)	1.64 (4)	2.560 (2)	152 (3)
N1—H1···O1	0.92 (3)	2.07 (3)	2.628 (2)	118 (2)
N1—H1···O2^i^	0.92 (3)	2.09 (3)	2.892 (2)	146 (3)

Symmetry codes: (i) −*x*+1, −*y*+2, *z*+1/2.
